# Early Predictors of Learning a Foreign Language in Pre-school – Polish as a First Language, English as a Foreign Language

**DOI:** 10.3389/fpsyg.2018.01813

**Published:** 2018-09-25

**Authors:** Marta Łockiewicz, Zuzanna Sarzała-Przybylska, Małgorzata Lipowska

**Affiliations:** Institute of Psychology, Faculty of Social Sciences, University of Gdańsk, Gdańsk, Poland

**Keywords:** early predictors, English as a foreign language, pre-school, Polish, oral English skills

## Abstract

When a foreign language (FL) acquisition begins in preschool, at which time young learners are particularly linguistically sensitive, it allows for a higher FL competence in future. Moreover, a second language learning depends on a learner’s aptitude. The aim of our study was to assess the early predictors of learning English as a Foreign Language (EFL) in Polish pre-school students who had not yet started formal literacy instruction, and to characterize the level of their oral receptive and active skills in English. 30 children aged between 3 years 5 months and 5 years 10 months who attended two private and one state kindergarten, participated in the study. All were native speakers of Polish, and apart from English classes, communicated in their first language at kindergarten and in their everyday life. Non-verbal intelligence, emerging literacy, phonological awareness in Polish, and knowledge of English were assessed. We found that in Polish pre-school children emerging letter identification from their first language alphabet, phonological awareness in their first language, and non-verbal intelligence were related to the achievements in learning EFL, despite the differences in transparency between the two languages. Moreover, the children’s passive color vocabulary was larger than their active vocabulary, and they were used to repetition tasks. The participants in our study attempted to communicate in English during the assessment, which suggests that even at a pre-school age they were able to differentiate between first language and FL discourse. We also identified some problems possibly stemming from linguistic transfer, like articles omissions. Therefore, teachers should pay more emphasis to the differences between the first and the second language, in terms of: syntax, morphology, phonetics, phonology, and orthography, to prevent later consolidation of early errors. The automatisation of correct linguistic habits in young learners would equip them with skills for their later FL educational success.

## Introduction

In Poland, learning English as a foreign language (EFL) combines sequential and subordinative acquisition (Kubiak, 2003), as formal instruction begins relatively early at pre-school entry (optionally and due to parental decision at 3 years of age, obligatory at 6 years of age, into Reception Year, cf. Regulation of the Minister of National Education, 2014 Journal of Laws, item 803). Thus, an EFL instruction begins before or simultaneously with literacy instruction in L1. This educational situation creates an opportunity to examine the acquisition of a new language system once the learners have an already relatively extensive knowledge about language as such (Kohnert et al., 2010), but may still use a native language (L1) acquisition strategies for a foreign language (FL) learning (Olpińska-Szkiełko, 2015).

Language development involves the learning of symbols and of rules that govern them, which is reflected in phonological, morphological-syntactic, semantic, and pragmatic skills (Krasowicz-Kupis, 2008). According to the core curriculum published by the Ministry of National Education, children at school entry (generally at 7 years of age) should: be interested in and ready to read, write, and spell, listen to stories and fairy tales and discuss them, be able to segment simple words into sounds and syllables, orally express their understanding of the world, recognize some letters and read short words appearing in everyday activities, experiment with language, tell stories and answer questions, classify objects into categories based on their size, shape, usage (Regulation of the Minister of National Education, 2017 Journal of Laws, item 356, attachment No. 1). Thus, in Polish pre-schools (entry at 3 years of age, due to parental decision; obligatory at 6 years of age), speaking skills and print awareness are developed (Krasowicz-Kupis et al., 2015b), as a preparation for literacy instruction, which is based on an analytic-synthetic teaching strategy (Awramiuk and Krasowicz-Kupis, 2014), combined with a global one (Jaszczyszyn, 2010).

This type of literacy instruction is adjusted to the characteristics of Polish language. Polish orthography, as compared to English, is more transparent, regular, and consistent in its grapheme-phoneme correspondence (Awramiuk and Krasowicz-Kupis, 2014). Though both languages follow the Subject – Verb – Object pattern in affirmative sentences, Polish syntax is more flexible. In fact, the aforementioned word order is only preferable, not mandatory, as a syntactic function of a word is indicated by its morphology (suffixes and inflections: declension and conjugation) (Polański and Nowak, 2011). In Polish, there are no articles, no inversion to formulate questions, implied subject may be used, and words are, on average, longer than English ones (Jaskulska and Łockiewicz, 2017).

The pre-school environment is an artificial condition for learning a FL in a culturally influenced social context. This is especially conspicuous in Poland, which is a largely monolingual country (National Census of Population and Housing, and Central Statistical Office of Poland, 2011). Thus, the learners have limited opportunities to communicate in a FL outside school, where, in addition, a FL exposure is limited to a FL class, while all other instruction and informal communication is conducted in Polish. Kersten and Rhode (2013) suggest that a pre-school routine should provide the most natural conditions, as this approach improves learning achievements. Moreover, the aims of teaching should include the development of positive motivation, exposure to foreign speech, and creating bases for systematic linguistic work (Komorowska, 2009). The core curriculum for preschools published by the Ministry of National Education in Poland states that an aim of pre-school education is to prepare children to use a modern FL through, among others, arousing language awareness and cultural sensitivity while playing games. Preschoolers at school entry should participate in plays, understand and follow simple instructions, repeat and sing nursery rhymes, understand the general point of short stories if their telling/reading by a teacher is accompanied with pictures, gestures, etc. Teachers should instruct children in a FL while at play, read them stories, use nursery rhymes, poems, songs, and audio–visual materials in a FL, to provide auditory, pre-literacy contact with a FL in different everyday situations (Regulation of the Minister of National Education, 2017 Journal of Laws, item 356, attachment No. 1). Thus, teaching strategies are based on interactive plays (Komorowska, 2005), and implicit, fun-focused techniques (Aguirre et al., 2016). As a result, the young learners should demonstrate specific skills of understanding commands, using simple phrases and nursery rhymes, and understanding the context of stories (Regulation of the Minister of National Education, 2017 Journal of Laws, item 356, attachment No. 1). Suggested methods of instruction encourage the EFL pre-school teachers to provide the young learners with an intensive contact with a FL, without the need for immediate oral production by the students (Kondrat, 2015). Kondrat (2015) suggests that all instructions should be given in English, children should learn by repeating, and through participation in science and art projects, using Content and Language Integrated Learning approach.

An early introduction of EFL teaching stems from evidence for its effectiveness. Olpińska-Szkiełko (2015) claims that if a FL acquisition begins in preschool, when young learners are particularly linguistically sensitive, it allows for a higher FL competence in future.

According to Cummins (1991), a second language (L2) learning depends on both the linguistic exposure quality and quantity and a learner’s aptitude. According to the linguistic interdependence theory, the development of L2 competence stems from the competence already developed in the first language (L1) at the time when L2 exposure begins (Cummins, 1979). Similarly, The Linguistic Coding Differences Hypothesis states that L1 skills provide the basic foundation for learning a FL (Ganschow and Sparks, 2000), phonological competence in particular (Sparks et al., 2012).

The predictive role of L1 phonological processing skills at a pre-literacy stage for EFL achievement has been reported in few studies. Phonological awareness allows to differentiate and manipulate phonological elements (Melby-Lervag et al., 2012). Children at first manipulate smaller, then larger phonological elements, which is influenced by schooling (Lipowska, 2001; Melby-Lervag et al., 2012). Syllable and intrasyllabic element awareness precedes letter identification (Awramiuk and Krasowicz-Kupis, 2014), while phonological sensitivity and letter knowledge reciprocally contribute to the development of one another prior to formal reading instruction (Burgess and Lonigan, 1998). In 5-year old Chinese children, syllable awareness predicted word reading at ages 8 and 10 (Pan et al., 2011), and in 5–6-year old Norwegian native speakers phonological awareness predicted spelling, word reading, and translation at age 11 (Helland and Morken, 2016) in learning EFL. In a bit older 6-year old English native speakers, at an early literacy stage, reading readiness (as measured with, e.g., phonological awareness tasks: rhyming and letter-sound relationships) predicted L2 (Spanish, French, and German) proficiency in Year 10 (Sparks et al., 2006). These studies outlined the importance of the identification of early predictors of a FL; they, however, measured the actual FL proficiency at a later stage of education, when the learners had already received literacy instruction, not the relationship between these early predictors and FL oracy skills in preschool. We intended to add to the existing literature by providing a simultaneous assessment of phonological processing in L1 and EFL skills before the formal literacy instruction began.

The aim of our study was to assess the early predictors of learning EFL in preschool students who had not yet started formal literacy instruction and to characterize the level of their oral receptive and active EFL skills. We assumed that phonological processing and literacy skills, specifically letter identification in L1, would be linked to the development also in FL oral language skills, following the line of thought in Sparks et al. (2006). We also aimed to examine how the young learners respond to the current teaching methodology, by the description and analysis of their actual performance, as compared with the expected one, outlined in the core curriculum. A unique group of participants took part in our study: preschoolers who take EFL classes in a monolingual country. We decided to examine such a young group, as, according to a new Polish legislation, English instruction will be obligatory in all kindergartens. Moreover, Sparks et al. (2006) reported, L1 predictors of students’ oral L2 skills change over time. Therefore, we decided to examine both the L1 predictors and the oral L2 skills at pre-school age. As non-verbal IQ and age of L2 acquisition are sometimes included as moderators in L2 acquisition studies (cf. a meta-analysis by Melby-Lervåg and Lervåg, 2011), we decided to control for these variables also in a EFL study.

To our knowledge, our study is a first attempt to assess the results of a pre-school curriculum for EFL in Poland using FL skill’s measures consistent with the curriculum.

## Materials and Methods

### Materials

(1) *Raven’s Colored Progressive Matrices –* a Polish adaptation (Szustrowa and Jaworowska, 2003) – assesses the level of non-verbal intelligence. Reliability for 4;5 – 4;11 years old children: *r*_tt_ = 0.75, *SEM* = 1.87. The validity correlation with *WISC test* for 7–9 years old children: *r* = 0.48–0.57. The test was administered in Polish.
(2) *Letter Naming Test* from *IBE Reading Tests Battery BTCZ IBE* (Krasowicz-Kupis et al., 2015a) – assesses Polish alphabet knowledge. Children name letters printed on a board. Score was 1 point for every correct answer, *Min* = 0, *Max* = 32. Reliability for 5;6 – 5;11 years old children: *r_tt_* = 0.96, *SEM* = 0.37. The validity correlation (Spearman’s rho) with *Vocabulary Test for Children* (Koć-Januchta, 2013): *r* = 0.54. The test was administered in Polish. We decided to use this test even though formal literacy instruction does not take place in Polish preschools. However, some children are nevertheless informally instructed, and 6-year-olds attempt to refer letters to sounds (Awramiuk and Krasowicz-Kupis, 2014).
(3) *Phonological Tests Battery* *BTF IBE* (Krasowicz-Kupis et al., 2015c) – assess phonological awareness skills. Score was 1 point for every correct answer. Discontinuation rule: five consecutive errors and/or lack of answer, with the exception of a *Phonemic hearing* task: no discontinuation rule. We administered the following tasks:
  (a) *Phonemic hearing* (based on non-words) – minimal pairs comparison (*Max* = 25 points).
  (b) *Alliterations – non-words* – comparison of two non-words starting with the same/different letter (*Max* = 16 points).
  (c) *Rhymes – recognition, words* – identification of a word that does not rhyme with two other rhyming words from a set of three. An auxiliary pictorial material was used (*Max* = 12 points).
  (d) *Syllables – blending, non-words* – repetition of a heard non-word segmented into syllables (*Max* = 10 points).

In calculations, we used a total composite score tapping phonological awareness (*Max* = 63 points. Reliability for 5;6 – 5;11 years old children: *r*_tt_ = 0.88, *SEM* = 0.72. The validity correlation (Spearman’s rho) with *Vocabulary Test for Children* (Koć-Januchta, 2013): from *r* = 0.42 to *r* = 0.49. The test was administered in Polish.

(4) *English Knowledge Test* – Łockiewicz, not published^1^. Score was 1 point for every correct answer; there were no discontinuation rules. The test was administered mostly in English. The tasks were based on the core curriculum skills for kindergartens as outlined by the Ministry of National Education in Poland, and included (the researcher’s questions and instructions are marked with italics):
  (a) A greeting: a child had to respond to a greeting (a greeting: *Hello*), introduce themselves (a question: *What is your name?)*, and give their age (a question: *How old are you?*) (*Max* = 3 points).
  (b) Color recognition: indicating the colors named by the researcher, e.g., *Point to red (pink, yellow)* (*Max* = 3 points)^2^.
  (c) Color naming: naming the colors (green, white, blue) pointed to by the researcher (a question: *What color is this?)* (*Max* = 3 points) (see footnote text 2).
  (d) Animal naming: naming the animals (a dog, a bird, a bear) pointed to by the researcher (a question: *What is it?)* (*Max* = 3 points) (see footnote text 2).
  (e) Phrase repetition: repeating three phrases (a big cow, an old man, a red car) said by the researcher (an instruction: *Repeat*) (*Max* = 3 points).
  (f) Following instruction: drawing an apple (an instruction: *Draw an apple*) (*Max* = 1 point). The children were given a paper and a pencil to complete the task.
  (g) A nursery rhyme 1. repetition: The researcher sung a popular nursery rhyme *The wheels on the bus*. The child had to repeat the single lines recited by the researcher (*Max* = 7 points), 2. comprehension: The child had to answer the questions (questions: *What is the song about?*, *What do the wheels do?, How long do the wheels go?)* about the nursery rhyme (*Max* = 3 points). The comprehension questions were asked first in English, then, if a child did not answer, in Polish. Both answers in English and in Polish were accepted, if correct semantically. No pictorial auxiliary material was used, though gestures were (e.g., circling moves to imitate wheels).

In calculations, we used a total composite score tapping the knowledge of English (*Max* = 26 points). All tasks are based on oral language skills, and supported with pictorial material, as the preschoolers had not yet started formal literacy instruction, even in their NL, according to the state-wide core curriculum. Reliability: *r*_tt_ = 0.89, *SEM* = 1.22. The correlations (Spearman’s rho) of subscales with total score were: greeting *r* = 0.658, color recognition *r* = 0.624, color naming *r* = 0.594, animal naming *r* = 0.756, phrase repetition *r* = 0.739, drawing (following instructions) *r* = 0.698, nursery rhyme – repetition *r* = 0.666, nursery rhyme – comprehension *r* = 0.717 (all *p* ≤ 0.001).

### Participants

30 children aged between 41 (3 years 5 months) and 70 (5 years 10 months) months (*M* = 54.93, *SD* = 8.29), who attended two private and one state kindergarten, participated in the study. 19 (63%) girls and 11 (37%) boys were matched for gender [χ^2^(19) = 17.8, *p* = 0.536], age [*M*_girls_ = 54.42, *SD*_girls_ = 8.14, *M*_boys_ = 55.82, *SD*_boys_ = 8.88; *t*(28) = 0.44, *p* = 0.664], and intelligence [*M*_girls_ = 16.63, *SD*_girls_ = 3.91, *M*_boys_ = 16.64, *SD*_boys_ = 4.24; *t*(28) = 0.00, *p* = 0.998]. All children were native speakers of Polish, and attended the same, 30-min long classes taught by the same teacher. In 1 pre-school, English was taught three times a week, in two pre-schools – once a week, in the morning. On average, the children had studied English for 2 years (*M* = 1.97, *SD* = 1.41, *Min* = 1.00, *Max* = 3.00). All other activities, classes, and communication with teachers were conducted in Polish. No homework was assigned. However, 5 (16.67%) children attended private English classes.

### Procedure

All assessments were carried out by the second author at the pre-school. During the first session, non-verbal intelligence and emerging literacy in Polish were assessed. During the second session, phonological awareness and knowledge of English were assessed. Each of the sessions lasted about 20 min. The protocol was approved by the Ethics Board for Research Projects at the Institute of Psychology, University of Gdańsk, Poland. Prior to the study, written informed consent was obtained from the participants’ parents. All children gave their oral consent to participate.

## Results

### English Oral Skills in Polish Preschoolers

**FIGURE 1:**
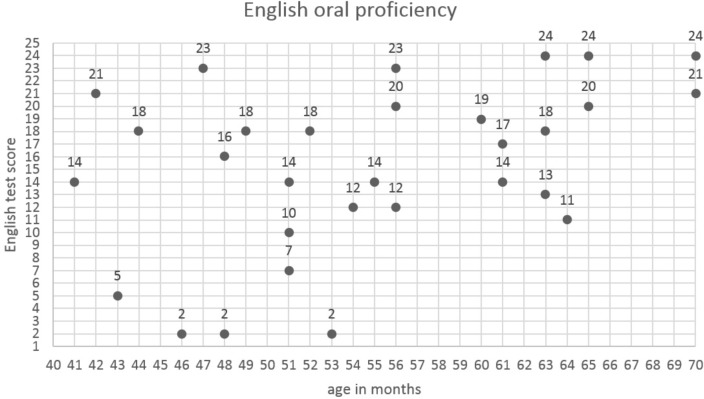
English oral skills according to age – the participants’ individual scores.

The descriptive statistics for letter identification and phonological awareness in Polish, and English oral skills and phonological awareness skills in Polish pre-school children are presented in **Table 1**. The actual scores in English oral skills of each child organized according to age are presented in **Figure 1**. As literacy instruction had not yet started in the pre-school, a small number of children actually knew any letters. For example, 15 children (50%) received letter identification scores of 0 or 1, and 18 children (60%) scores of 0, 1, or 2.

**Table 1:** Letter identification, phonological awareness, non-verbal IQ, and English oral skills in Polish pre-school children – raw scores.

	*M*	*Mdn*	*Min*	*Max*	*SD*	*SKE*	*K*
Letter identification	4.47	1.50	0.00	25.00	7.05	2.01	3.16
Phonological awareness	35.63	34.00	18.00	59.00	10.92	0.18	–0.94
Non-verbal IQ	16.63	16.50	10.00	25.00	3.97	0.42	–0.27
**English oral skills**		
Greeting	2.17	2.00	1.00	3.00	0.83	–0.03	–1.49
Color recognition	2.03	2.00	0.00	3.00	0.93	–0.62	–0.11
Color naming	1.30	1.00	0.00	3.00	1.18	0.31	–1.40
Animal naming	0.77	1.00	0.00	3.00	0.86	0.84	–0.11
Phrase repetition	2.37	3.00	0.00	3.00	1.07	–1.55	1.01
Drawing (following instruction)	0.43	0.00	0.00	1.00	0.50	0.28	–2.06
Nursery rhyme: repetition	5.27	7.00	0.00	7.00	2.61	–1.36	0.34
Nursery rhyme: comprehension	0.93	0.00	0.00	4.00	1.36	1.00	–0.70
Total score	15.20	16.50	2.00	24.00	6.66	–0.63	–0.39


In our study, the children’s age correlated positively and moderately with English oral skills, *r* = 0.394, *p* = 0.031. Accordingly, two oldest children scored the highest, 24 (92%) and 21 (81%) points, respectively. However, the two youngest children scored 14 (54%) and 21 (81%) points, respectively. 3 (10%) children achieved the highest score of 24 (92%) points, and 3 (10%) children achieved the lowest score of 2 (8%) points. The scores distribution was negatively skewed, but close to normal, showing that the English test was not too difficult for the children (Shapiro–Wilkes’s coefficient was *p* = 0.040).

Below we present the level of English oral skills in relation to particular tasks.

**FIGURE 2:**
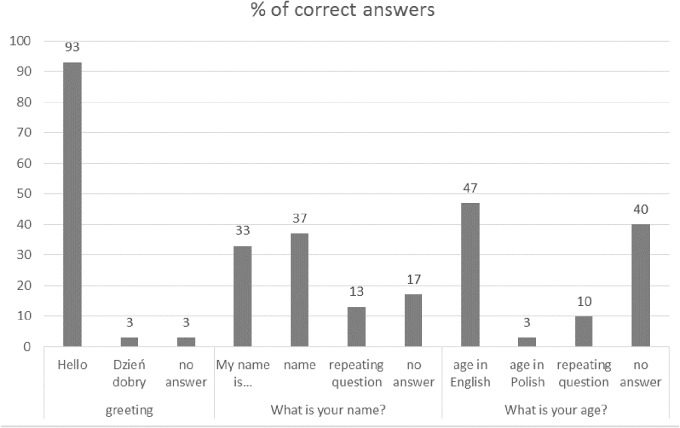
Polish pre-school children’s answers to demographic questions in English.

Greeting: 13 children (43%) scored 3 points, i.e., they repeated *Hello* to the examiner’s greeting, gave their name, and age, 9 (30%) – 2 points, and 8 (27%) – 1 point. Specifically, 28 (93%) children responded: *Hello*, 1 (3%) –*Dzień dobry* (*Good morning* in Polish), and 1 (3%) gave no answer. 10 (33%) children responded with a full sentence: *My name is*…, 11 (37%) gave only their name, 4 (13%) repeated the researcher’s question, 5 (17%) gave no answer. 14 (47%) children gave their age, 1 (3%) gave their age in Polish, 3 (10%) repeated the researcher’s question, 12 (40%) gave no answer or an incorrect answer (**Figure 2**).

**FIGURE 3:**
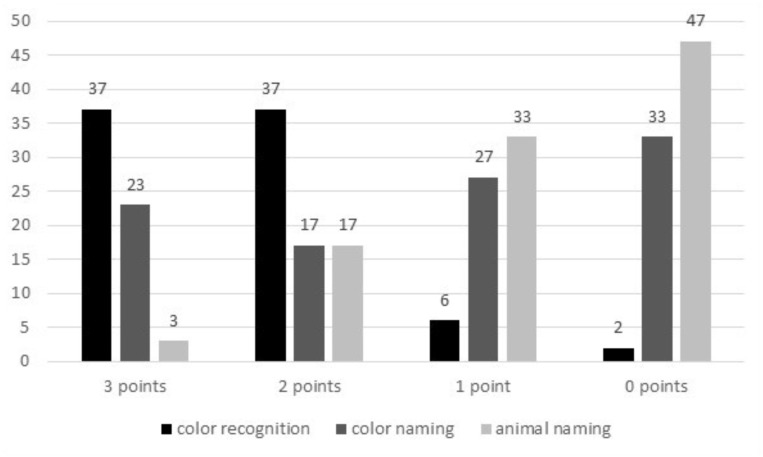
Polish pre-school children’s color recognition and naming, and animal naming in English.

Color recognition: 11 (37%) children scored 3 points, 11 (37%) – 2 points, 6 (20%) – 1 point, 2 (7%) – 0 points (**Figure 3**). Specifically, 25 (83%) preschoolers recognized red color, 22 (73%) – pink, 15 (50%) – yellow. 1 (3%), 3 (10%), and 4 (13%) children gave the incorrect names of colors, respectively.

Color naming: 7 (23%) children scored 3 points, 5 (17%) – 2 points, 8 (27%) – 1 point, 10 (33%) – 0 points (**Figure 3**). Specifically, 14 (47%) preschoolers named green color, 9 (30%) – white, and 18 (60%) – blue. 2 (7%), 0 (0%), and 1 (3%) children gave the incorrect names of colors, respectively. A Wilcoxon Signed-Ranked test indicated that the children’s passive color vocabulary was better than their active vocabulary, *Z* = 3.513, *p* ≤ 0.001, *r* = 0.46.

Animals naming: 1 (3%) child scored 3 points, 5 children (17%) – 2 points, 10 (33%) – 1 point, 14 (47%) – 0 points (**Figure 3**). 13 (43%) preschoolers named *a dog* (in addition, 1 (3%) child used a Polish word: *pies*), 3 (10%) – *a bird*, and 7 (23%) – *a bear* (including 5 (17%) who knew the specific species: *a polar bear*; in addition, 1 (3%) child used a Polish word: *niedźwiedź*).

**FIGURE 4:**
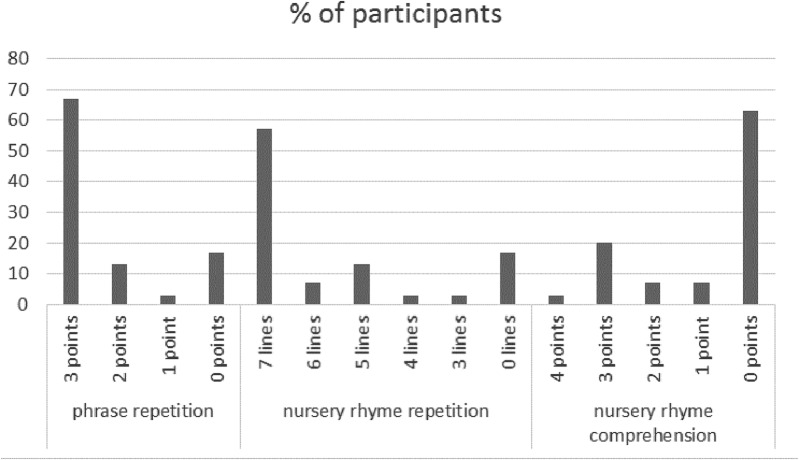
Polish pre-school children’s phrase repetition and nursery rhyme repetition and comprehension in English.

Phrase repetition task: 20 (67%) children scored 3 points, 4 (13%) – 2 points, 1 (3%) – 1 point, 5 (17%) – 0 points (**Figure 4**). 25 (83%) preschoolers repeated *a big cow* [including, however, 2 (7%) who omitted the indefinite article], 22 (76%) repeated *an old man* [including, however, 10 (35%) who either omitted or distorted the indefinite article], and 22 (73%) repeated *a red car* [including, however, 5 (17%) who either omitted or distorted the indefinite article]. In total, approximately half (16 children) of 27 preschoolers who attempted the task (10%, i.e., 3 children, failed to repeat even 1 phrase) either omitted, or distorted an article.

Nursery rhyme repetition: 17 (57%) children repeated all 7 lines, 2 (7%) – 6 lines, 4 (13%) – 5 lines, 1 (3%) – 4 lines, 1 (3%) – 3 lines, and 5 (17%) – 0 lines. Nursery rhyme comprehension: 1 (3%) child scored 4 points, 6 (20%) children – 3 points, 2 (7%) – 2 points, 2 (7%) – 1 point, 19 (63%) – 0 points (**Figure 4**).

Instruction following: 13 (43%) children drew an apple.

### Early Native Language Predictors of English as a Foreign Language Oral Skills

The Polish language tests scores positively correlated moderately, strongly, and very strongly with English oral language skills. Letter identification correlated with age (*r* = 0.624, *p* ≤ 0.001), non-verbal IQ (*r* = 0.293, *p* = 0.058, statistical trend) and English skills, total score (*r* = 0.394, *p* = 0.031). Phonological awareness measured on Polish words and non-words correlated with age (*r* = 0.531, *p* = 0.003), non-verbal IQ (*r* = 0.379, *p* = 0.019) and English skills, total score (*r* = 0.559, *p* ≤ 0.001). English oral language skills also correlated with age (*r* = 0.394, *p* = 0.031) and non-verbal IQ (*r* = 0.518, *p* = 0.002).

Two hierarchical multiple regression analyses were conducted (**Table 2**). Age was entered as an independent variable in Step 1, non-verbal IQ was entered as an independent variable in Step 2, and phonological awareness and letter identification skills in L1 were entered as an independent variable in Step 3, respectively.

**Table 2:** Results of two hierarchical regression analyses in which age, non-verbal IQ, and either phonological awareness or letter identification skills in L1 were regressed upon English oral language skills of Polish pre-school children.

Predictor	English oral language skills		
	β	*t*	*p*
**Step 1**			
Age	0.414	2.41	0.023*
Δ*R*^2^	0.172*		
**Step 2**			
Age	0.194	1.02	0.316
Non-verbal IQ	0.415	2.18	0.038*
Δ*R*^2^	0.124*		
**Step 3 for L1 phonological awareness**
Age	0.026	0.13	0.898
Non-verbal IQ	0.363	1.20	0.056^a^
L1 phonological awareness	0.374	2.07	0.049*
Δ*R*^2^	0.099*		
Total *R*^2^/Adj. *R*^2^	0.395/0.325^∗∗^		
**Step 3 for letter identification**
Age	0.015	0.08	0.939
Non-verbal IQ	0.392	2.22	0.035*
Letter identification	0.403	2.37	0.025*
Δ*R*^2^	0.125*		
Total *R*^2^/Adj. *R*^2^	0.421/0.354^∗∗^		


*^∗∗^p ≤ 0.01, ^∗^*p* ≤ 0.05, ^*a*^statistical trend.*

The first regression analysis for English oral language skills showed that the independent variables: age, non-verbal IQ, and phonological awareness explained a total of 33% of the variance (*F*_3,26_ = 5.66, *p* = 0.004). Significant independent variables in Step 3 were: non-verbal IQ (β = 0.363, statistical trend) and phonological awareness based on material in Polish (β = 0.374). The second regression analysis showed that the independent variables: age, non-verbal IQ, and letter identification explained a total of 35% of the variance (*F*_3,26_ = 6.29, *p* = 0.002). Significant independent variables in Step 3 were: non-verbal IQ (β = 0.035) and Polish letter identification (β = 0.424) (see **Table 2**). In both models, an apparent prediction of age (cf. Step 1 in **Table 2**) disappeared when non-verbal IQ was added.

## Discussion

In our study, the children’s age and non-verbal IQ correlated positively and moderately with English oral receptive and active skills, which is an expected result due to the learners’ linguistic and cognitive development and assumed longer FL education. However, the two youngest children scored 54 and 81% of all possible points, respectively. This result supports the notion of early commencement of a FL education. Hidaka et al. (2012) concluded that the neural foundation for a FL processing could be established at a developmental stage (during 3–5 years of age) after some length of linguistic exposure. In their research, the brain activity in the bilateral frontal areas of 5-year-old Japanese native speakers who had been exposed to English for 2 years (exposure for at least 15 min per day in their first year, and approximately for 4 h per day in their second year of pre-school instruction) was higher for both their L1 and EFL, as compared with the activity for a rarely exposed L3 (Chinese), and consistent with that in Japanese adults. No difference in the brain activity for different languages was observed in 4-year olds who had been exposed to English for 1 year only. Conversely, Lecumberri and Gallardo (2003) reported that early introduction to formal non-natural exposure to the FL does not facilitate FL sound acquisition, as students who started English instruction at age 8 were better in vowel and consonant discrimination than students who started at age 4 (similar to the age of our participants who started formal English instruction at the age of 3), after identical number of teaching hours. This could be due to cognitive maturity of the learners and different teaching methods used in school as compared with kindergarten. In our study, we did not assess sound discrimination.

In our study, despite attending the same preschools and having been taught by the same teachers, the participants differed substantially in their English oral skills. 10% of children scored 92% of all possible points, while another 10% – only 8%. This disparity might be due to the features of the FL teaching (e.g., the instructed vocabulary, different teaching methodology) and testing methods.

We found that phonological awareness in L1 and non-verbal IQ (statistical trend) predicted English oral language skills of Polish pre-schoolers learning EFL, but apart from EFL class generally instructed in L1, when age and non-verbal IQ were controlled for. Moreover, we found that letter identification, limited to Polish alphabet, and non-verbal IQ predicted English oral language skills of Polish pre-schoolers learning EFL, when age and non-verbal IQ were controlled for. These findings are consistent with a report of a cross-linguistic transfer of oral language and phonological awareness skills for learning English as L2, in a different context of partial immersion (schooling in L2 for at least 4 h per day) (Melby-Lervåg and Lervåg, 2011). It must be remembered, though, that L2 acquisition differs much from a FL acquisition, as the latter is characterized by much less exposure to linguistic content, less intensive FL instruction, and fewer options for daily, authentic, and meaningful communication practice. Nevertheless, our results suggest that the transfer happens also in case of a limited exposure. L1 phonological awareness, especially syllable and phoneme awareness, predicted EFL decoding abilities in 5–6-year old Korean children, who were instructed in English (Kang, 2012). Similarly, L1 phonological awareness of 5–6-year old pre-literacy, Year 1 pre-school Norwegian students predicted their subsequent EFL spelling, word reading, and translation skills when 11-year old (Helland and Morken, 2016). Thus, a cross-linguistic transfer of phonological skills occurs in educational contexts of different FL exposure. However, Chinese tone awareness, but not rhyme awareness, predicted EFL word reading and phonological awareness in 4–6-year old Cantonese native speakers, which suggests a different level of L1 phonological processing impact (Yeung and Chan, 2013). Therefore, more evidence from other pairs of languages is needed.

The aforementioned findings are consistent with the Linguistic Coding Differences Hypothesis which states that L1 acquisition skills relate to FL learning skills, due to the phonological code (Sparks et al., 2012), and that skills in L1 provide the basic foundation for learning a FL (Ganschow and Sparks, 2000). In Petrus and Bogdanowicz’s (2004), research an opposite direction of relation was examined: how EFL skills influence L1 acquisition. The authors found that 4–5-year old Polish preschoolers who learned EFL scored higher in a task assessing L1 rhyme recognition (which is developed earlier in English than in Polish (cf. Stanovich et al., 1984; Krasowicz-Kupis, 1999) as compared with their peers who did not participate in an EFL class. However, both groups performed on a level in a L1 alliteration recognition task, and monolinguals outperformed bilinguals in L1 phoneme discrimination, which was interpreted as evidence for a phonological system common for both languages. Thus, a relation between phonological awareness in L1, FL, and L2 seems to be consistently evidenced in different pairs of languages, both for oral FL, as our study demonstrated, and for literacy skills.

However, we noticed that also non-verbal IQ predicted English oral language skills in Polish pre-schoolers learning EFL. This is consistent with reports of the influence of non-verbal IQ on L2 learning aptitude (Grigorenko et al., 2000; Brooks et al., 2017).

Cenoz (2003) reported that after 600 h of learning EFL, students who started learning English as L3 (L1 – Basque, L2 – Spanish) at age 4, were less proficient than those who started at age 8 and 11, likely due to cognitive maturity and less developed test taking strategies.

As long-term memory is important in FL vocabulary acquisition (Cheung, 1996; Masoura and Gathercole, 2005), the process of a FL teaching should involve systematic rehearsals and automatisation (Woźnicki and Zawadzka, 1981), which is an achievable task in young learners. Over 1/3 of our participants introduced themselves with a full sentence, which demonstrated that they had been taught to repeat/respond with entire phrases. Almost half of the group gave their age and over 40% drew an apple following the researcher’s instruction (this task had been included to check for comprehension without the need for a verbal answer, which could have been more difficult due to limited FL lexicon), which indicated comprehension. This finding was supported by an observation that only in three cases did the preschoolers use a Polish word instead of an English one. However, each time the response was semantically correct, demonstrating that the children understood the questions, but lacked vocabulary. Moreover, in the apple drawing task it was possible for the participants to perform the task based on the lexical knowledge of the word *apple* only, without understanding the instruction itself, as they were handed a piece of paper and a pencil when instructed. Hence, the performance in this task might in fact add more to the color/animal naming task lexical skill assessment, rather than instruction following. Moreover, the participants in our study attempted to communicate in English, which suggests that even at a pre-school age they were able to differentiate between L1 and FL discourse. Cenoz (2003) reported that the early introduction of EFL (L3) at age 4 is not associated with a higher level of language confusion, as compared with the introduction at age 8 or 11, for example using the interlocutor’s choice of language as a clue. In fact, Singleton (2003) claims that the majority of L2 researchers agree that an early and, in particular, substantial exposure to L2 is related to a higher FL proficiency that a later one (starting in adolescence or later), even though he does not support the idea of a critical period in a FL acquisition. For example, younger children are more likely to produce words based on fixed, learnt patterns, as compared with older children, who more often employ such strategies as over-generalization, reading pronunciation, or pronunciation guessing, due to cognitive maturation (Lecumberri and Gallardo, 2003). Bialystok and Hakuta (1999), as cited in: Marinova-Todd (2003) claim that young children’s unstable, still developing knowledge of L1, interferes less with their learning of an L2.

In addition, our finding corroborates suggestions for teachers to converse with young learners solely in L2 (cf. Kondrat, 2015). Olpińska-Szkiełko (2015) summarizes research reports that *early total immersion programs are most effective* (p. 207); however, this would require for the children to attend an English-language pre-school, which was not the case in our study, and is a rare situation in Polish educational system. Our results show, nevertheless, that pre-school teachers may successfully conduct and expect communication with their students in L2 only even if all of them share the same L1 that is used for other instruction and in everyday life.

We also found that the children’s passive color vocabulary was larger than their active vocabulary, as they indicated more colors than they named, which is consistent with literature (Laufer, 1998). For all three colors, half or more of the children recognized each of them individually, which suggest that the children had learnt color vocabulary. Interestingly, 2 out of 3 of these terms are much shorter in English, than in Polish (1-syllable *red* and *pink*, and 2-syllable *yellow*, as compared with corresponding 3-syllable *czerwony* and *różowy*, and 2-syllable *żółty*). They failed to name them, though, as only less than 50% of the participants recalled 1 of 3 given colors. In this case, all these terms are much shorter in English, than in Polish (1-syllable *green*, *white*, and *blue*, as compared with corresponding 3-syllable *zielony*, 2-syllable *biały*, and 3-syllable *niebieski*). Similarly, less than 50% of the participants named 1 of 3 given animals. Here the length of words was more similar (1-syllable *dog*, *bird*, and *bear*, as compared with corresponding 1-syllable *pies* and *ptak*, and 2-syllable *niedźwiedź*, the latter word, however, has a much more complex syllable structure).

A majority of preschoolers repeated all or almost all single lines of a nursery rhyme and all three phrases (an article+an adjective+a noun) said by the researcher, which demonstrates that they are used to repetition tasks. In few cases, they even repeated the researcher’s words when not supposed to. Teacher repetition is also important in FL instruction, as it allows for, e.g., students’ recognition and practice of a target language item, as evidenced in the work with Korean preschoolers with minimum EFL skills (Roh and Lee, 2018). However, we accepted as correct a phrase that consisted of an adjective and a noun only, as approximately half of preschoolers who attempted the task either omitted, or distorted an article. These omissions might result from a negative linguistic transfer (Odlin, 1989; Zybert, 1999), as pre-schoolers largely ignored articles which do not occur in Polish. Therefore, teachers should concentrate on types of students’ FL learning difficulties due to the linguistic transfer, to prevent the occurrence and consolidation of incorrect linguistic habits and increase accuracy (Lewandowska, 2013). In case of differences between L1 languages that have no determiners, such as Polish, and EFL, the teachers should emphasize the use of non-transferable structures and rules, e.g., through always presenting a new word and/or phrase with its highlighted article. The children’s frequent omission of grammatical morphemes could also be attributed to a transitional developmental stage frequently observed in younger children’s first language acquisition and early FL production where sentences are produced in a “telegraphic” form including only (or mostly) content words and no (or few) function words (cf. Carroll, 2008; Fromkin et al., 2011). It would be interesting to conduct a longitudinal study in which the average order of acquisition of English grammatical morphemes in Polish pre-schoolers learning EFL, and its relation to the comparability of corresponding Polish structures and rules would be examined.

However, we found that when asked questions about the nursery rhyme, a majority of preschoolers failed to answer. As the participants repeated more lines than they comprehended, we assume that their phonemic hearing, which allows for sound discrimination, has been developed correctly (Petrus and Bogdanowicz, 2004), and manifested also in discrimination of non-native sounds and their combinations. In early partial immersion programs, which are advocated in many early education textbooks, children usually develop at first receptive skills (listening comprehension) (Olpińska-Szkiełko, 2015). In future projects, we would like to include in the study methods a survey of the teachers teaching approach in this respect.

## Limitations

The major limitation of our study is the use of a non-validated measure for assessing an FL oral skills, due to a lack of available standardized instruments. However, when designing the screening tool, we based the questions and tasks on the core curriculum for the pre-school. Moreover, we intend to conduct a follow-up study in which the participants will be additionally screened for language-related disabilities.

## Conclusion

Our study produced evidence-based knowledge about the results and the (individual and teaching) variables of learning an FL at pre-school, which is crucial given the current European investments in FL teaching and expectations about students’ achievement, as *the early introduction of (FLs) in kindergarten (…) has expanded in Europe* (Cenoz, 2003, p. 77). We found that in Polish pre-school children, at a pre-literacy level of education, emerging letter identification and phonological awareness, in their L1 were related to the achievements in learning English as a FL, despite the differences in transparency between the two languages. Yeung and Chan (2013) underlined *the importance of the L1 phonological awareness in L2 phonological awareness development that is a crucial building block for future reading development* (p. 563). Thus, we believe that support strategies for young learners who fail to acquire FL skills should include phonological awareness skills in both L1 and FL. We also identified some problems possibly stemming from linguistic transfer, like articles omissions. Therefore, teachers should pay more emphasis to the differences between L1 and FL syntax, morphology, phonetics, phonology, and orthography, to prevent later consolidation of early errors and promote correct linguistic habits, as focus on vocabulary is likely a necessary but insufficient approach (Lonigan et al., 2008). For example, young learners should practice full structures and sentences to reinforce correct patterns. This could happen if pre-school FL instruction, regardless of frequency and intensity of exposure, is implemented in conditions as similar as possible to L1 acquisition (Olpińska-Szkiełko, 2015), to provide a natural situational and communication context for linguistic interaction. Such methods include: the Narrative Format (Taeschner, 2005; Pirchio et al., 2015), the Accelerative Integrated Method,^3^ or the Good Start Method for English (Bogdanowicz et al., 2015). A list of educational tools and useful websites with tips designed for Polish pre-school teachers of EFL, is also provided in a publication by Kondrat (2015). The automatisation of correct linguistic habits in young learners would equip them with skills for their later FL educational success.

## Author Contributions

MŁ designed the study and the data collection protocol, developed hypotheses, conducted statistical analyses, analyzed and interpreted data. ZS-P collected data as part of her fulfillment of the M.A. in Psychology. MŁ and ZS-P drafted the manuscript. ML provided critical revisions of the manuscript.

## Conflict of Interest Statement

The authors declare that the research was conducted in the absence of any commercial or financial relationships that could be construed as a potential conflict of interest.

## Footnotes

1 Full text of the test and data underlying the study available from the corresponding author upon request.

2 We used 2 pictures for these tasks: 1. for color repetition and naming, and 2. for naming and counting animals, with the permission of the author: Ewa Grzybowska.

3 https://www.aimlanguagelearning.com/media/
